# Affordance Equivalences in Robotics: A Formalism

**DOI:** 10.3389/fnbot.2018.00026

**Published:** 2018-06-08

**Authors:** Mihai Andries, Ricardo Omar Chavez-Garcia, Raja Chatila, Alessandro Giusti, Luca Maria Gambardella

**Affiliations:** ^1^Institute for Systems and Robotics (ISR-Lisboa), Instituto Superior Técnico, Lisbon, Portugal; ^2^Istituto Dalle Molle di Studi sull'Intelligenza Artificiale, USI-SUPSI, Lugano, Switzerland; ^3^Institut des Systèmes Intelligents et de Robotique, Sorbonne Université, Centre National de la Recherche Scientifique, Paris, France

**Keywords:** affordance, learning, cognitive robotics, symbol grounding, affordance equivalence

## Abstract

Automatic knowledge grounding is still an open problem in cognitive robotics. Recent research in developmental robotics suggests that a robot's interaction with its environment is a valuable source for collecting such knowledge about the effects of robot's actions. A useful concept for this process is that of an affordance, defined as a relationship between an actor, an action performed by this actor, an object on which the action is performed, and the resulting effect. This paper proposes a formalism for defining and identifying affordance equivalence. By comparing the elements of two affordances, we can identify equivalences between affordances, and thus acquire grounded knowledge for the robot. This is useful when changes occur in the set of actions or objects available to the robot, allowing to find alternative paths to reach goals. In the experimental validation phase we verify if the recorded interaction data is coherent with the identified affordance equivalences. This is done by querying a Bayesian Network that serves as container for the collected interaction data, and verifying that both affordances considered equivalent yield the same effect with a high probability.

## 1. Introduction

Symbolic grounding of robot knowledge consists in creating relationships between the symbolic concepts used by algorithms controlling the robot and the physical concepts to which they correspond (Harnad, 1990). An *affordance* is a concept that allows collection of grounded knowledge. The notion of *affordance* was introduced by Gibson (1977), and refers to the action opportunities provided by the environment. In the context of robotics, an affordance is a relationship between an actor (i.e., robot), an action performed by the actor, an object on which this action is performed, and the observed effect.

A robot able to discover and learn the affordances of an environment can autonomously adapt to it. Moreover, a robot that can detect equivalences between affordances can quickly compute alternative plans for reaching a desired goal, which is useful when some actions or objects suddenly become unavailable.

**Figure 1:**
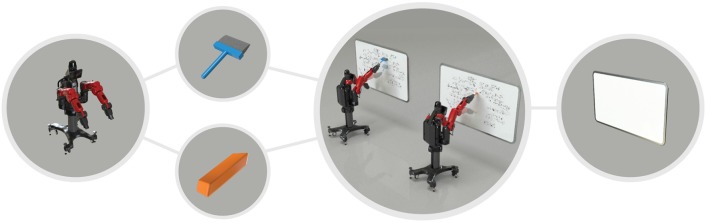
Example of equivalence between two objects for cleaning a whiteboard: a wiper and an eraser. The robot affords to clean the white board by wiping it either with a wiper or an eraser.

**Figure 2:**
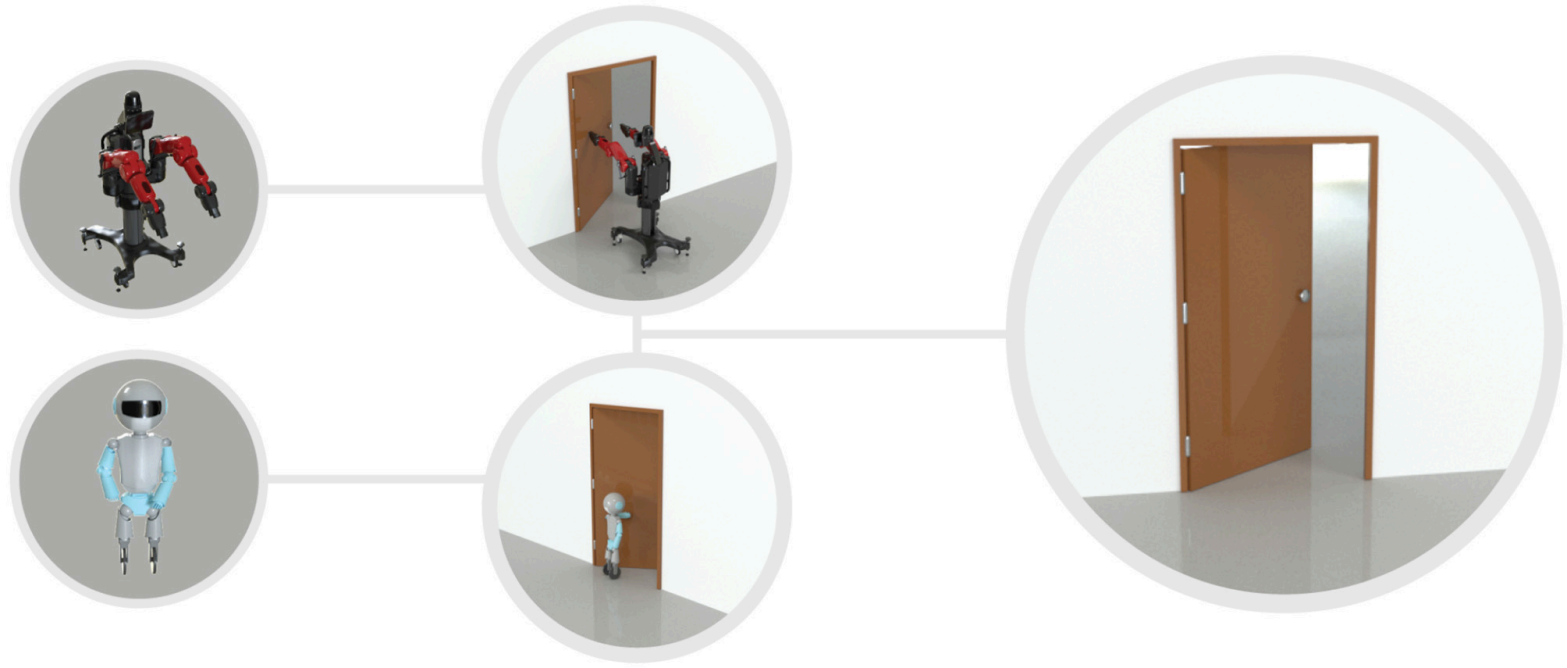
Example of equivalence between different actors and their actions for opening a door. A door can be opened by any robot that can interact with the door.

In this paper, we introduce a method for identifying affordances that generate equivalent effects (see examples in Figures 1, 2). We define a (comparison) operator that allows robots to identify equivalence relationships between affordances by analysing their constituent elements (i.e., actors, objects, actions).

### 1.1. Affordance discovery and learning

All methods proposed in the literature for affordance learning are similar in viewing an interaction as being composed of three components: an action, a target object, and a resulting effect. Different methods were proposed to infer the expected effect, given knowledge about the action and target object.

Several papers approached affordance learning as learning to predict object motion after interaction. For this purpose, Krüger et al. (2011) employed a feedforward neural network with backpropagation which learned so-called *object-action complexes*; Hermans et al. (2013) used Support Vector Machines (SVM) with kernels; while Kopicki et al. (2017) employed Locally Weighted Projection Regression (LWPR) with Kernel Density Estimation and a mixture of experts. Ridge et al. (2009) first used a Self-Organising Map and clustering in the *effect space* to classify objects by their effect, and then trained a SVM which identified to which cluster an object belongs using its feature-vector description.

Other papers addressed affordance learning from the perspective of object grasping. Stoytchev (2005) employed detection of invariants to learn object grasping affordances. Ugur et al. (2012) used SVMs to study the traversability affordance of a robot for grasping. Katz et al. (2014) used linear SVM to learn to perceive object affordances for autonomous pile manipulation. More details on the use of affordances for object manipulation can be found in the dissertation of Hermans (2014).

Some works followed a supervised training approach, providing hand-labeled datasets which mapped objects images (2D or RGB-D) to their affordances. Myers et al. (2015) learned affordances from local shape and geometry primitives using Superpixel-based Hierarchical Matching Pursuit (S-HMP), and Structured Random Forests (SRF). Image regions (from RGB-D frames) with pre-selected properties were tagged with specific affordance labels. For instance, a surface region with high convexity was labeled as *containable* (or a variation of it). Varadarajan and Vincze (2012) proposed an Affordance Network for providing affordance knowledge ontologies for common household articles, intended to be used for object recognition and manipulation. An overview of machine learning approaches for detecting affordances of tools in 3D visual data is available in the thesis of Ciocodeica (2016).

Another approach for learning affordances uses Bayesian Networks. Montesano et al. (2008) and Moldovan et al. (2012) employed a graphical model approach for learning affordances, using a Bayesian Network which represents objects/actions/effects as random variables, and which encodes relations between them as dependency links. The structure of this network is learned based on the data of robot's interaction with the world and on *a priori* information related to the dependency of some variables. Once learned, affordances encoded in this way can (1) predict the effect of an action applied to a given object, (2) infer which action on a given object generated an observed effect, and (3) identify which object generates the desired effect when given a specific action.

Yet another popular method for supervised affordance learning uses Deep Learning techniques. For instance, Nguyen et al. (2016) trained a convolutional neural network to identify object affordances in RGB-D images, employing a dataset of object images labeled pixelwise with their corresponding affordances. A similar approach using a deep convolutional neural network was taken by Srikantha and Gall (2016).

Recent comprehensive overviews of affordance learning techniques are available in the dissertation of Moldovan (2015), and in reviews by Jamone et al. (2016), Min et al. (2016), and Zech et al. (2017).

We argue that once affordances are learned, we can find relations between affordances by considering the effects they generate. One of these relations is equivalence, i.e., when two different affordances specify corresponding actions on objects that generate the same effect.

### 1.2. Affordance equivalence

Affordance equivalence was studied by Şahin et al. (2007), who considered relationships between single elements of an affordance. Thus, it was possible to identify objects or actions that are equivalent with respect to an affordance when they generate the same effect. Griffith et al. (2012) employed clustering to identify classes of objects that have similar functional properties. Montesano et al. (2008) and Jain and Inamura (2013) treated affordance equivalence from a probabilistic point of view, where, in the context of imitation learning, the robot searches for the combination of action and effect that maximises their similarity to the demonstrated action on an object. Boularias et al. (2015) discovered through reinforcement learning the graspability affordance over objects with different shapes, and indirectly showed equivalence of the grasp action.

Developing this line of thought, we propose a probabilistic method to identify which *combinations of affordance elements* generate equivalent effects. We first present in section 2 the affordance formalization employed, and based on that we then list in section 2.4 all the possible types of affordance equivalences.

Since the purpose of this study is to identify equivalences between affordances that were already recorded by the robot, we are not seeking to explain how to record these affordances. In this paper we employed the graphical model approach for learning affordances proposed by Montesano et al. (2008). In addition, we rely purely on perception-interaction data, without using *a priori* information (Chavez-Garcia et al., 2016b). To facilitate the experimental setup, we used pre-defined sensorial and motor capabilities for our robots.

The remainder of this paper is organized as follows. In section 2, we introduce our formalization of affordance elements, and define the equivalence relationship in section 2.4. A series of experiments on the discovery of equivalences between affordances is detailed in section 3, together with the obtained results. We conclude and present opportunities for future work in section 4.

## 2. Methodology: affordance formalization

In this section, we present the affordance formalism employed throughout the paper. We follow the definition proposed by Ugur et al. (2011), that we enrich by including the actor performing the action into the affordance tuple *(object, action, effect)*. The inclusion of the actor into the affordance allows robots to record affordances specific to their body morphologies. Although we will not focus on this aspect in this paper, it is possible to generalize this knowledge through a change of affordance perspective from robot joint space to object task space (more about this in section 2.1.2).

**Figure 3:**
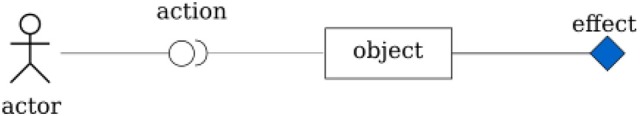
A graphical representation of an affordance. An object accepts any action that fits its interface (shown on object's left), and produces the specified effect (shown on object's right). Any actor capable of performing the expected action on this object can produce the described effect.

We define an affordance as follows. Let *G* be the set of actors in the environment, *O* the set of objects, *A* the set of actions, and *E* the set of observable effects. Hence, when an *actor* applies an *action* on an *object*, generating an *effect*, the corresponding affordance is defined as a tuple:

(1) $$\begin{array}{r} \alpha =\left(actor,object,action,effect\right),\text{ for }actor\in G,object\in O,\\ \;action\in A,\text{ and }effect\in E, \end{array}$$

and can be graphically represented as shown in Figure 3. From actor perspective, it interacts with the environment (the object) and discovers the affordances. From object perspective, affordances are properties of objects which can be perceived by actors, and which are available to actors with specific capabilities. We can also consider observers, who learn by perceiving other actors' affordance acquisition process.

The way in which affordance elements are defined influences the operations that can be performed with affordances. Since we aim to establish equivalence relationships between affordances, we will analyse the definitions of the following affordance elements: actions (from actor and object perspectives), objects (as perceived by robot's feature detectors), and effects (seen as a description of the environment).

### 2.1. How are actions defined?

Actions can be defined (1) relative to actors, by describing the body control sequence during the execution of an action in joint space; or (2) relative to objects, by describing the consequences of actions on the objects in operational space. We refer to *object perspective* when the actions are defined in the operational/task space, making their definition independent of the actor executing them. We refer to *actor perspective* when the actions are defined in the joint space of the actor, making them dependent of the actor executing them.

This statement comes from the different perspectives obtained from the affordance definition in Equation (1): *actor* and *object* perspective.

#### 2.1.1. Actions described relative to actors

Actions are here described relative to actors and their morphology. They are defined with respect to their control variables in joint space (i.e., velocity, acceleration, jerk), indexed by time τ:

(2) $$\begin{array}{r} action:{\left\{Q,\overset{\cdot }{Q},\ddot{Q}\right\}}_{\tau } \end{array}$$

As the action is described with respect to the actor morphology and capabilities, comparing two actions requires comparing both the actors performing the actions, and the actions themselves. When the actors are identical, the action comparison is straightforward. However, when there is a difference between actors' morphologies (and their motor capabilities), the straightforward comparison of actions is not possible and a common frame of reference for such comparison is needed.

#### 2.1.2. Actions described relative to objects

When actions are described relative to objects, they represent an action generalisation from the *joint space* of a particular actor (where actions are defined on the actor) to the *operational space* of any actor (where actions are defined on the object).

Thus, when actions are described relative to objects, the *actor* can be omitted from the affordance tuple, to indicate that any actor which has the required motor capabilities is able to generate the action which causes this effect. In addition, the action employed in this representation is defined in *operational space* (and not in *joint space* as before). Hence, dropping the actor from the equation, we can rewrite Equation (1) as:

(3) $$\begin{array}{r} \alpha =\left(object,action,effect\right),\text{ for }object\in O,action\in A_{o},\text{ and }\\ \;effect\in E \end{array}$$

where *A*_*o*_ is the set of all actions in operational space, applicable to object *o*.

While affordances defined from actor perspective (in joint space, e.g., joint forces to apply) allow to learn using robot's motor and perceptual capabilities, affordances defined from object perspective (in task space, e.g., forces applied on the object) allow to generalise this knowledge.

### 2.2. How are objects defined?

If an actor has the feature detectors *p*_1_, …, *p*_*n*_ corresponding to its perception capacities (such as hue, shape, size), then an object is defined as:

(4) $$\begin{array}{r} object=\left\{p_{1},\ldots ,p_{n}\right\}, \end{array}$$

where each feature detector can be seen as function on a perceptual unit (e.g., a salient segment from a visual perception process).

### 2.3. How are effects defined?

We suppose that an actor *g* has a set ξ of *q* effect detectors, that are able to detect changes in the world after an action *a* ∈ *A*_*g*_ is applied. For example, when an actor executes action *push* on an object, the object-displacement-effect detector would be a function that computes the difference between two measurements of the object position taken before and after the interaction. Another effect can be the difference in the feedback force measured in the end effector before and after the interaction. Formally, effects are a set of *q* salient changes in the world ω (i.e., in the target object, the actor, or the environment), detected by robot's effect detectors ξ:

(5) $$\begin{array}{r} effect=\left\{\xi_{1}\left(\omega \right),\ldots ,\xi_{q}\left(\omega \right)\right\} \end{array}$$

### 2.4. Affordance equivalence operator

In this section, we introduce the concept of affordance equivalence, based on the formalization presented earlier in section 2. We provide truth tables for two different affordance comparison operators: one for the case where actions are defined in actor joint space, and one for the case where actions are defined in object task space. For each case, we explore the possible types of affordance equivalence.

We have defined an affordance as a tuple of type (*actor, object, action*_*joint_space*_, *effect*) when the action is defined relative to the actor, or as a tuple of type (*object, action*_*operational_space*_, *effect*) when the action is defined relative to the object. Let us now define the truth table for an operator for comparing affordances (one for the actor perspective, and one for the object perspective) and identifying equivalence relationships between them.

*We consider equivalent two affordances that generate equivalent effects*. To know when two effects are equivalent, an effect-comparison function is required. We define an equivalence function *f*(*e*_*a*_, *e*_*b*_) that yields true if two effects values *e*_*a*_ and *e*_*b*_ are similar in a common frame (e.g., distances for position values, similarity in color models, vector distances for force values). We detect affordance equivalence by (1) feeding the continuous (non-discretised) data on the measured effects to the Bayesian Network (BN) structure learning algorithm, and then (2) querying the BN over an observed effect to obtain the empirical decision on effect equivalence. Whenever two affordances generate equivalent effects, it is possible to find which affordance elements cause this equivalence. We distinguish several cases of affordance equivalence, depending on the elements which differ in two equivalent affordances, which are detailed below.

#### 2.4.1. Equivalence between affordance with actions defined relative to actors

The comparison cases for affordances with actions described relative to actors are shown in Table 1. The 2^4^ cases of comparison between the elements of two affordances stem from all the possible (binary) equivalence combinations between the elements. In each case we compare the four components and establish if the elements of affordances are equivalent.

**Table 1:** Comparison of two affordances, when actions are described with respect to actors.

**#**	**Actors**	**Objects**	**Actions**	**Effects**	**Conclusion**
1	different	different	different	different	*(actor, object, action)* non-equivalence
2	different	different	different	equivalent	***(actor, object, action)*** **equivalence**
3	different	different	same	different	*(actor, object)* non-equivalence
4	different	different	same	equivalent	***(actor, object)*** **equivalence**
					
5	different	same	different	different	*(actor, action)* non-equivalence
6	different	same	different	equivalent	***(actor, action)*** **equivalence**
7	different	same	same	different	*actor* non-equivalence
8	different	same	same	equivalent	***actor*** **equivalence**
					
9	same	different	different	different	*(object, action)* non-equivalence
10	same	different	different	equivalent	***(object, action)*** **equivalence**
11	same	different	same	different	*object* non-equivalence
12	same	different	same	equivalent	***object*** **equivalence**
					
13	same	same	different	different	*action* non-equivalence
14	same	same	different	equivalent	***action*** **equivalence**
15	same	same	same	different	impossible in deterministic systems
16	same	same	same	equivalent	**due to determinism**

*Equivalence cases between affordances are presented in even rows. Inconsistencies are underlined in red. The types of affordance equivalence are shown in bold letters*.

Since actions are defined here relative to the actors, actors with different morphologies cannot perform the same action defined in joint space, because their joint spaces are different. This renders inconsistent cases in which different actors perform the same action: lines (3), (4), (7), and (8) in Table 1. This leaves us with five cases of equivalence in Table 1, where:

- If different actors using different actions on different objects generate an equivalent effect, then we have *(actor, action, object) equivalence*
- If different actors using different actions on the same object generate an equivalent effect, then we have *(actor, action) equivalence*
- If the same actor using different actions on different objects generates an equivalent effect, then we have *(object, action) equivalence*
- If the same actor using the same action on different objects generates an equivalent effect, then we have *object equivalence*
- If the same actor using different actions on the same object generates an equivalent effect, then we have *action equivalence*.

We assume that the environment is a deterministic system: each time the same actor applies the same action on the same object, it will generate an equivalent effect. Therefore, generating a different effect with the same actor, action, and object is impossible, due to determinism.

Both the effect equivalence and non-equivalence cases provide information about the relationship between two affordances. The affordance equivalence concept is empirically validated in section 3.

#### 2.4.2. Equivalence between affordances with actions defined relative to objects

The comparison cases for affordances with actions described relative to objects are shown in Table 2. There are 2^3^ cases of comparison, corresponding to the total number of possible (binary) equivalence cases between the elements of a pair of affordances. In this case, three types of equivalence exist:

- If different actions on different objects generate the same effect, then it is (*object, action*) equivalence;
- If same action on different objects generates the same effect, then it is *object* equivalence;
- If different actions on same object generate the same effect, then it is *action* equivalence.

**Table 2:** Comparison of two affordances, when actions are described with respect to objects.

**#**	**Objects**	**Actions**	**Effects**	**Conclusion**
1	different	different	different	*(object, action)* non-equivalence
2	different	different	equivalent	***(object, action)*** **equivalence**
3	different	same	different	*object* non-equivalence
4	different	same	equivalent	***object*** **equivalence**
					
5	same	different	different	*action* non-equivalence
6	same	different	equivalent	***action*** **equivalence**
7	same	same	different	impossible in deterministic systems
8	same	same	equivalent	**due to determinism**

*Equivalence cases between affordances are presented in even rows. The types of affordance equivalence are shown in bold letters*.

## 3. Experiments and results: affordance equivalence

We designed experiments that would confirm the capability of our affordance representation to detect equivalences and non-equivalences between learned affordances. We employed a Bayesian Network structure-learning approach presented in (Chavez-Garcia et al., 2016a) to describe and learn affordances as relations between random variables (affordance elements). Then we analyse how the learned affordances relate to each case of equivalence presented in Table 2.

### 3.1. Pre-defined actions

We assume that an agent is equipped, since its conception, with motor and perceptual capabilities that we called *pre-defined*. However, we do not limit the agent's capabilities to the pre-defined set, as through learning the agent may acquire new capabilities. In our scenario, we employed three robotic actors of different morphologies, each with its pre-defined actions:

1. Baxter_gripper_: the Baxter robot's left arm (7 DOF) equipped with a gripper, with actions:
  - Push (moving with constant velocity without closing the gripper)
  - Pull (closing the gripper and moving with constant velocity)
  - Wipe (closing the gripper and pressing downwards while moving)
  - Move aside (closing the gripper and moving aside)
2. Baxter_nogripper_: the Baxter robot's right arm with no gripper, with action:
  - Poke (moving forwards with constant acceleration)
3. Katana arm with no gripper (5 d.o.f.), with action:
  - Side push (moving aside with constant velocity)

**Figure 4:**
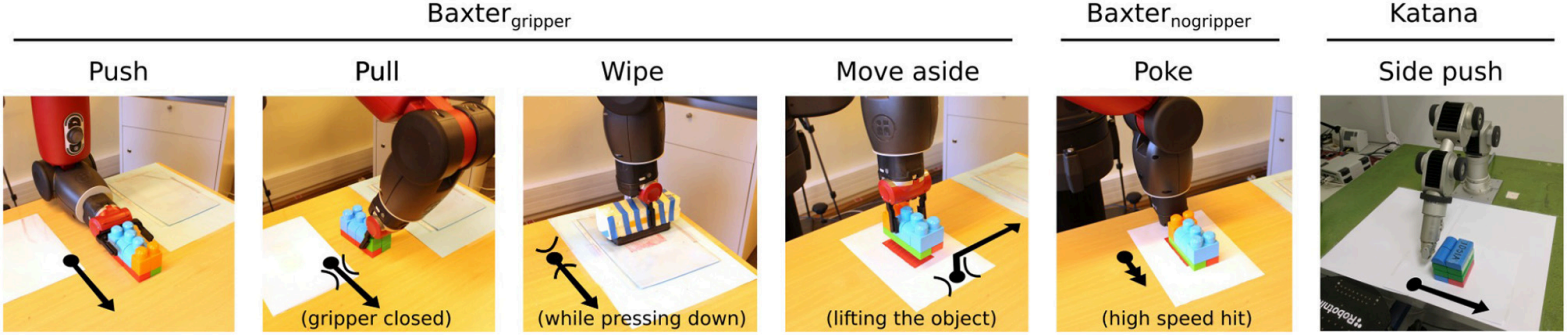
Set of pre-defined actions for three actors: Baxter_gripper_ equipped with a 7 d.o.f. arm, and an electrical gripper attached to it, Baxter_nogripper_ equipped only with a 7 d.o.f. arm, and Katana 5 d.o.f. arm without gripper. Poke is the only pre-defined action of actor Baxter_nogripper_, and side push the only pre-defined action of Katana. The arrows show the direction of the manipulator movement. The arcs show the position of the gripper with respect to the object, while the black bullet represents the object.

The actors and their pre-defined sets of actions (motor capabilities) are shown in Figure 4.

### 3.2. Pre-defined perceptual capabilities

**Figure 5:**
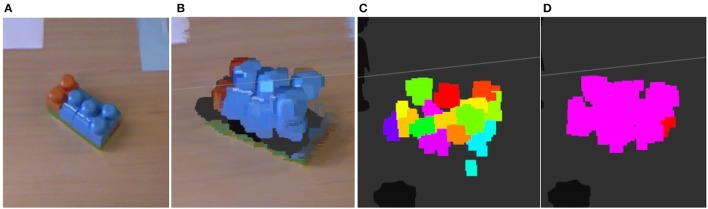
An example of the visual perception process output. From left to right: **(A)** reference image **(B)** RGB cloud of points of the scene **(C)** supervoxel extraction **(D)** clusterization of supervoxels. For visual perception we use a Microsoft Kinect sensor that captures RGB-D data.

Our visual perception process takes raw RGB-D data of an observed scene to oversegment the point cloud into a supervoxel representation. This 3D oversegmentation technique is based on a flow-constrained local iterative clustering which uses color and geometric features from the point cloud (Papon et al., 2013). Strict partial connectivity between voxels guarantees that supervoxels cannot flow across disjoint boundaries in 3D space. Supervoxels are then grouped to obtain object clusters that are used for extracting features and manipulation. Figure 5 illustrates the visual perception process. The objects employed were objects of daily use: toys that can be assembled, markers, and dusters. The objects were selected so as to be large enough to allow easy segmentation and manipulation.

### 3.3. Pre-defined effect detectors

**Figure 6:**
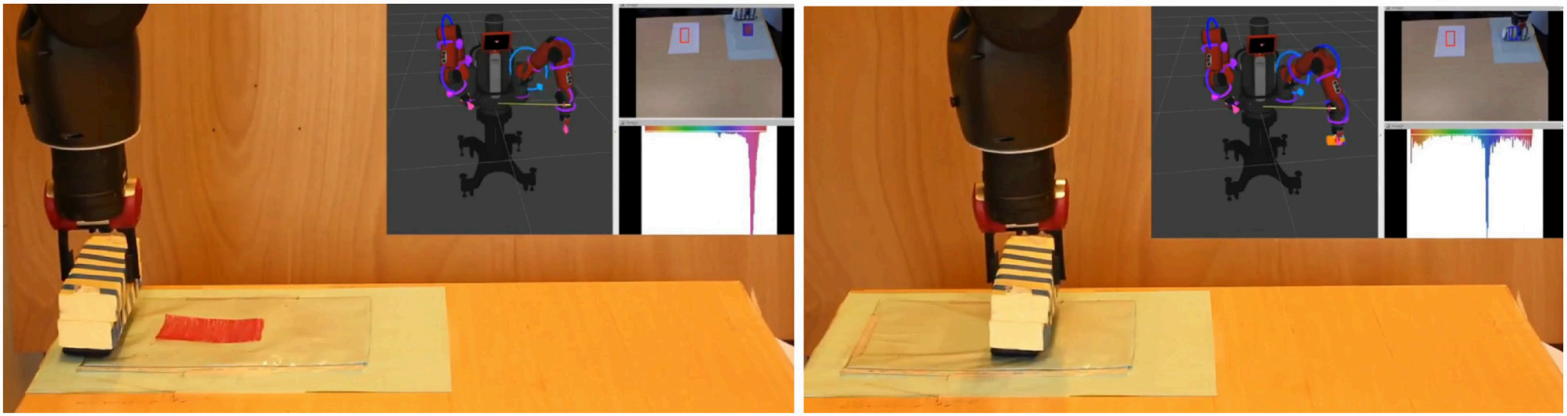
Example of captured effects when performing the action *wipe* on the object *duster*. Left figure shows the spatial (pose) and perceptual (color) state of the *duster*, and the *surface*. After *wipe* action is performed, the effects on position and in hue are detected: *duster* has changed position but not color, *surface* has changed color but not position. Although for this experiment we do not use the force in the joints, we are also capturing these changes.

We used custom hand-written effect detectors for the experimental use-cases, although our experimental architecture allows for an automatic effect detector. An effect detector quantifies the change, if present, in one property of the environment or the actor. For this series of experiments, we developed the following effect detectors: color change in a 2D image (HSV hue) for an object or a region of interest; object's position change (translation only); and the end-effector position. Figure 6 illustrates the detected effects when *wipe* action is performed. In our previous work we covered changes in joint torques, distance between finger grippers and object speed.

### 3.4. Affordance learning

Affordance elements *E* (effects), *O* (objects) and *A* (actions) are represented as random variables of a Bayesian Network (BN) B. First, in each actor interaction we record the values (discretized) for the random variables representing the objects (section 3.2), actions (section 3.1), and effects (section 3.3). The problem of discovering the relations between *E*, *O*, and *A* can be then translated to finding dependencies between the variables in B, i.e., P(B|D) learning the structure of the corresponding network B from data D. Thus, affordances are described by the conditional dependencies between variables in B.

We implemented an information-compression score to estimate how well a Bayesian Network structure describes data D (Chavez-Garcia et al., 2016b). Our score is based on the Minimum Description Length (MDL) score:

(6) $$\begin{array}{r} MDL\left(B|D\right)=LL\left(B|D\right)-|B|\frac{logN}{2}, \end{array}$$

where the first term measures (by applying a log-likelihood score Suzuki, 2017) how many bits are needed to describe data D based on the probability distribution P(B). The second term counts the number of bits needed to encode B, where $\frac{log\left(N\right)}{2}$ bits are used for each parameter in the BN. We consider $\frac{log\left(N\right)}{2}$ as factor that penalizes structures with larger number of parameters. For a BN's structure B, its score is then defined as the posterior probability given the data D.

We implemented a search-based structure learning algorithm based on the hill-climbing technique, as we did in our previous work. As inputs, this algorithm takes values for the variables in *E*, *O*, and *A* obtained from robot's interaction. This procedure estimates the parameters of the local probability density functions (pdfs) given a Bayesian Network structure. Typically, this is a maximum-likelihood estimation of the probability entries from the data set, which, for multinomial local pdfs, consists of counting the number of tuples that fall into each table entry of each multinomial probability table in the BN. The algorithm's main loop consists of attempting every possible single-edge addition, removal, or reversal, making the network that increases the score the most the current candidate, and iterating. The process stops when there is no single-edge change that increases the score. There is no guarantee that this algorithm will settle at a global maximum, but there are techniques to increase its reaching possibilities (we use simulated annealing).

By using the BN framework, we are capable of displaying relationships between affordance elements. The directed nature of its structure allows us to approximate cause-effects relationships. It also handles uncertainty through the established probability theory. In addition to direct dependencies, we can represent indirect causation.

#### 3.4.1. Detection of affordance equivalence

Equivalence between two affordances can be identified by comparing their ability to consistently reproduce the same effect *e*, judging by the cumulated experimental evidence. The precise type of equivalence between two affordances, which tells which affordance elements' values are equivalent, can be identified by probabilistic inference on the learned BN. Inference allows to identify which *(actor, object, action)* configurations are more likely to generate the same effect. In practice, this inference is calculated through executing queries to the Bayesian Network, which allow to compute the probability of an event (in our case: the probability of an effect having a value between some given bounds) given the provided evidence data.

Queries have the following form: *P*(*proposition*|*evidence*) where *proposition* represents the query on some variable *x*, and *evidence* represents the available information for the affordance elements, e.g., the identity of the actor, the description of the action, and the description of the object. In the example of the robot pushing an object, the following query allows to compute the probability of the object displacement falling between certain bounds:

(7) $$\begin{array}{l} P((position>lower\;bound)\text{ and }(position<upper\;bound)\;|\\ \;actor=Baxter,action=push,object=block) \end{array}$$

After querying the learned BN with the corresponding elements from Tables 1, 2 as evidence, if two *(actor, object, action)* configurations have probabilities of generating an effect that are higher than an arbitrary threshold, then we consider both affordances equivalent:

(8) $$\begin{array}{r} \text{ if }P\left(e|{actor}_{1},{object}_{1},{action}_{1}\right)>\theta \\ \text{ and }P\left(e|{actor}_{2},{object}_{2},{action}_{2}\right)>\theta \\ \text{then }\left({actor}_{1},{object}_{1},{action}_{1}\right)\equiv \left({actor}_{2},{object}_{2},{action}_{2}\right) \end{array}$$

For our experiments, we empirically established the equivalence threshold θ = 0.85. The aforementioned querying process connects the learning and reasoning steps, and according to the current goal of an actor, it allows for an empirical threshold selection or an adaptive mechanism.

### 3.5. Experimental results

**Figure 7:**
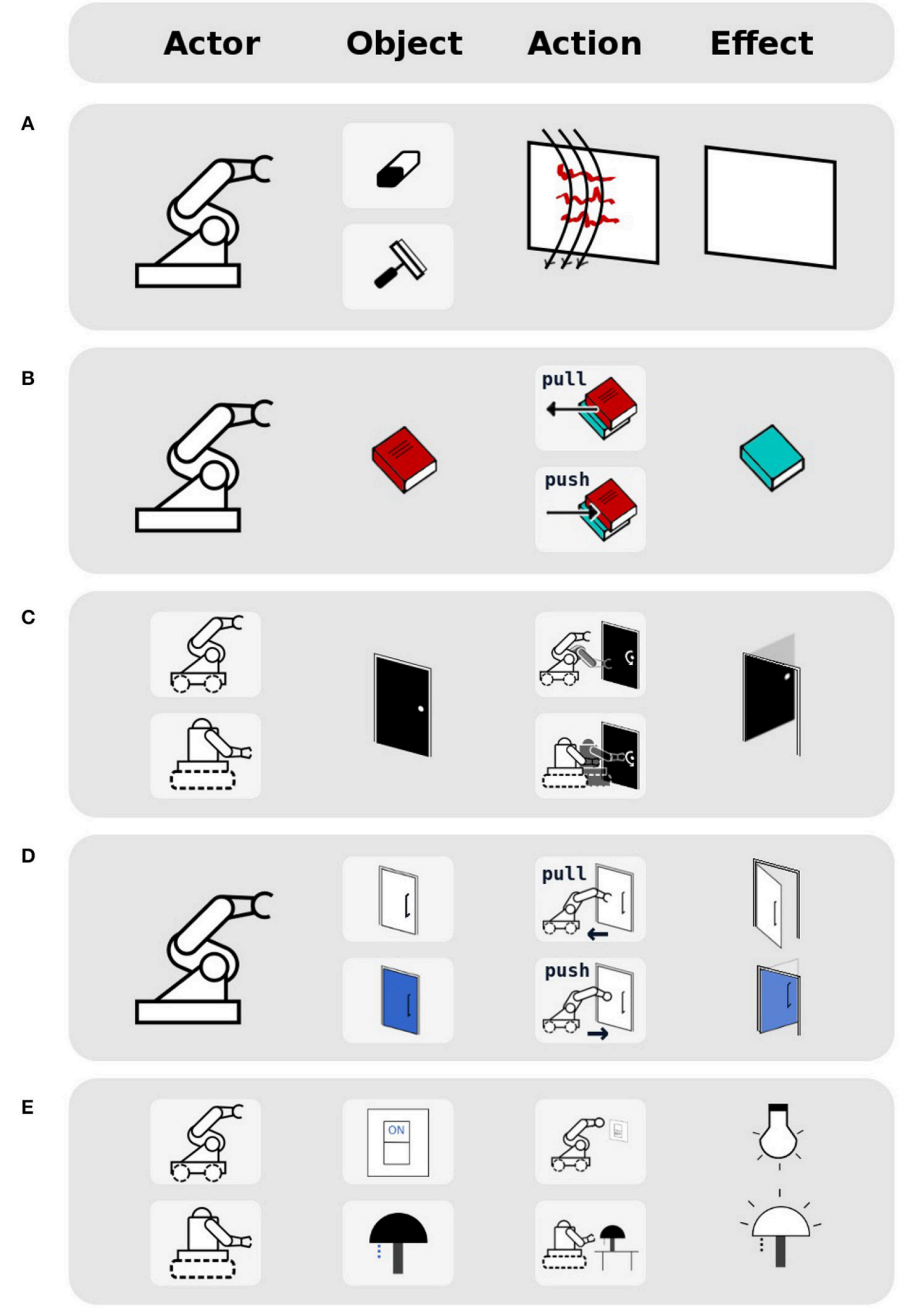
Illustrated examples for each of the five types of affordance equivalences, from the actor perspective, when affordances are represented as *(actor, object, action, effect)* tuples: **(A)** A robot can use two different objects (wiper/eraser) to obtain the same effect of obtaining a clean whiteboard when performing wipe action. **(B)** A robot can perform two different actions (push/pull) to obtain the same effect of revealing a book underneath. **(C)** Two different robots can perform two different actions on the same object to obtain the same effect of opening a door. **(D)** A robot can perform two different actions (pull/push) on two different objects (door handle/door) in order to obtain the same effect of opening those doors. **(E)** Two robots can apply two different actions on two different objects (light switch, lamp) to obtain the effect of turning on the light.

As shown in Table 1, affordances composed of 4 elements (actor, object, action, effect), which have their actions defined from the actor perspective, have five cases of equivalence (see Figure 7 for some illustrated examples). We have selected three of them to demonstrate the use of the affordance equivalence operator: (object) equivalence, (action) equivalence, and (actor, action) equivalence. In Figure 7 they correspond to the settings (a), (b), and (c). These experiments are detailed below. For a video demonstration of these experiments, please see the Supplementary Material section at the end of this document.

#### 3.5.1. The (actor, action) equivalence

This experiment consisted in discovering the equivalence between (actor, action) tuples. The goal was to identify configurations that are equivalent in their ability of uncovering a region of interest (a red mark on the table) by moving the object occluding it from robot's camera view (in the case of the Baxter — a toy with features color:blue and shape:box; in the case of the Katana actor — a box with the same perceptual features). In our representation, two objects with the same perceptual features are considered the same. Actor Baxter_gripper_ is equipped with a gripper and can perform action *move*_*aside*. Actor Baxter_nogripper_ does not have a gripper and can only perform action *poke*. Actor Katana does not have a gripper and can only perform *side push* action.

**Figure 8:**
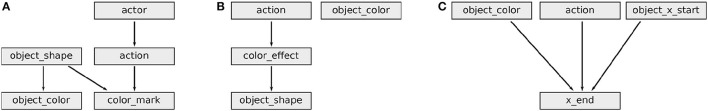
Learned Bayesian networks for the experiments. **(A)** (actor, action) **equivalence** between Baxter and Katana actors using different movements to reveal a colored region of interest (section 3.5.1). The BN shows the dependence between the chosen actors and actions and the revelation of the colored region of interest. **(B)** (object) **equivalence** between two dusters of different colors that clean a whiteboard (section 3.5.2). The BN shows the irrelevance of object_color feature for the wiping affordance. **(C)** (action) **equivalence** between push/pull actions (section 3.5.3). The BN shows the relation between the chosen action and the final displacement of the object (feature x_end).

The Bayesian Network structure was learned using data from 15 interactions using each (actor, action) tuple (Figure 8). Variables object_shape and object_color represent the object features, variable color_mark captures the presence or absence of a colored mark. Queries performed on the BN suggested that the effect of revealing the red mark is consistently recreated when moving the object toy, with a probability of 0.98 for the action move_aside done by the hand with a gripper, 0.97 for the action poke done by the hand with no gripper, and 0.94 for the action side_push done by the Katana arm on the box object. The probabilities are based on the total number of trials verifying these relationships. Since these affordances consistently recreate equivalent effects while having some equal elements (same toy object for Baxter_gripper_ and Baxter_nogripper_, and a similar object for Katana), this points that affordance elements that differ between configurations are in fact equivalent in their ability to generate the effect of revealing the red mark, i.e., the tuples (Baxter_nogripper_, poke), (Baxter_gripper_, move_aside) and (Katana, side_push). Source code of the experimental setup for the Katana actor is available at https://romarcg@bitbucket.org/romarcg/katana_docker.git.

#### 3.5.2. The (object) equivalence

The experiment consisted in determining the equivalence between two visually different whiteboard dusters: duster_blue_ and duster_orange_. Actor Baxter_gripper_ applies the same action wipe to remove a red marker trace from a blue colored surface, as shown in Figures 4, 6. For distinguishing the clean blue colored surface from the surface dirtied with the red marker, the robot's pre-defined effect detector measured the effect on the hue extracted from an HSV histogram.

The robot performed 25 trials of the *wipe* action with each duster, and the obtained data was subsequently used to learn the Bayesian Network structure (see Figure 8B). Objects are represented in the same way as in section 3.5.1. The effect capturing the change in the wiped area is described by the variable color_effect. Queries revealed that the wipe action cleans the red marker trace from the blue colored surface with a probability of 0.95 in both cases. Since the observed effects were equivalent, and the actor and action were the same, the objects duster_blue_ and duster_orange_ are considered equivalent in their ability to reach this effect.

#### 3.5.3. The (action) equivalence

In this experiment we analysed equivalence between the actions of an actor. This experiment consisted in placing the same object *toy* into a desired location using two different actions *push* and *pull* of the actor Baxter_gripper_. The robot performed 30 trials using each of the push and pull actions. Figure 8C shows the learned BN for (action) equivalence. The arrival of the object (described as in previous experiments) to the desired position is described by the effect variable x_end (only the x component of the 3D position was measured). The target location to which we aim to push/pull the object is at x coordinate 0.72 ± 0.02*m*. Variable object_x_start is an object feature representing the object initial position. According to the BN that processed the obtained data, there was a 0.97 probability to *pull* the object to the desired location, and a 0.89 chance to do so by *pushing* it. With all the rest being equal (the actor, object, and effect are the same), and since both actions have a high probability of generating the given effect, these *push* and *pull* actions can be considered equivalent for placing the object *toy* in a desired location.

## 4. Conclusions and future work

We have presented a formalization for affordances with respect to their elements, and the equivalence operator for comparing two affordances from the actor and object perspective. We performed Bayesian Network structure learning to capture affordances as sensorimotor representations based on the observed experimental data. We analysed and validated experimentally the affordance equivalence operator, demonstrating how to extract information on the tuples of actors, actions and objects by comparing two affordances and determining if such tuples are equivalent.

In practice, the learned affordance equivalences can be interchangeably used when some objects or actions become unavailable. In a multi-robot setting, these equivalences can allow an ambient intelligence (an Artificial Intelligence system controlling an environment) to select the appropriate robot for using an affordance to reach a desired effect.

### 4.1. Future work

Our future work will focus on the domain of transfer learning. We plan to implement a transformation between the affordances learned by specific robots (in their own joint space) to affordances applicable to objects and defined in their operational space. This will generalise the affordances learned and perceivable by a robot with a specific body schema, making them perceivable (and potentially available) to robots with any type of body schema (morphology).

We are already working on an automatic method for generating 3D object-descriptors. This would allow us to remove human bias from the way in which the robot observes and analyses its environment. By using an auto-encoder (a type of artificial neural network) that trains on appropriate datasets, it can automatically adapt to changes in objects that the robot interacts with.

Work is also underway on representing robot actions in a continuous space (e.g., using a vector representation of torque forces, or Dynamic Movement Primitives), which would be an improvement from today's discrete representation of actions (e.g., move, push, pull).

Ultimately, we intend to define an *algebra of affordances* detailing all the operations that are possible on affordances, and which would encompass operators such as affordance equivalence, affordance chaining (Ugur et al., 2011), and other operators that are still to explore.

## Author contributions

Literature review by MA and RC-G. Methodology and theoretical developments by MA, RC-G, and RC. Experiment design and implementation by MA and RC-G. Analysis of the experimental results by MA, RC-G, RC, AG, and LG. Document writing and illustrations by MA, RC-G, RC, AG, and LG.

### Conflict of interest statement

The authors declare that the research was conducted in the absence of any commercial or financial relationships that could be construed as a potential conflict of interest.
